# Reversing Age Related Changes of the Laryngeal Muscles by Chronic Electrostimulation of the Recurrent Laryngeal Nerve

**DOI:** 10.1371/journal.pone.0167367

**Published:** 2016-11-28

**Authors:** Michael Karbiener, Jonathan C. Jarvis, Justin D. Perkins, Hermann Lanmüller, Martin Schmoll, Hanna S. Rode, Claus Gerstenberger, Markus Gugatschka

**Affiliations:** 1 Department of Phoniatrics, ENT University Hospital, Medical University of Graz, Graz, Austria; 2 School of Sport and Exercise Sciences, Liverpool John Moores University, Liverpool, United Kingdom; 3 Department of Veterinary Clinical Sciences, Royal Veterinary College, London, United Kingdom; 4 Center of Medical Physics and Biomedical Engineering, Medical University of Vienna, Vienna, Austria; West Virginia University School of Medicine, UNITED STATES

## Abstract

Age related atrophy of the laryngeal muscles -mainly the thyroarytenoid muscle (TAM)- leads to a glottal gap and consequently to a hoarse and dysphonic voice that significantly affects quality of life. The aim of our study was to reverse this atrophy by inducing muscular hypertrophy by unilateral functional electrical stimulation (FES) of the recurrent laryngeal nerve (RLN) in a large animal model using aged sheep (n = 5). Suitable stimulation parameters were determined by fatiguing experiments of the thyroarytenoid muscle in an acute trial. For the chronic trial an electrode was placed around the right RLN and stimulation was delivered once daily for 29 days. We chose a very conservative stimulation pattern, total stimulation time was two minutes per day, or 0.14% of total time. Overall, the mean muscle fiber diameter of the stimulated right TAM was significantly larger than the non-stimulated left TAM (30μm±1.1μm vs. 28μm±1.1 μm, p<0.001). There was no significant shift in fiber type distribution as judged by immunohistochemistry. The changes of fiber diameter could not be observed in the posterior cricoarytenoid muscle (PCAM).

FES is a possible new treatment option for reversing the effects of age related laryngeal muscle atrophy.

## Introduction

In a rapidly ageing society, voice problems will become a major challenge within the near future. The population aged 65 years or over will account for 28.7% of the EU-28’s population by 2080, compared with 18.5% in 2014 [1]. The incidence of disordered vocal function in elderly patients in modern Western societies is usually estimated from 12 to 35% [2,3]. Weakening of the voice has long been neglected, but gained consideration lately, as vocal endurance is required in many professions up to higher ages. The term *presbyphonia* refers to various symptoms of vocal aging, while *presbylarynx* denotes the typical age related morphological changes observed during laryngoscopical examinations. Patients typically complain of increased vocal roughness, vocal instability, a shortening in phonation duration and a deterioration of voice quality throughout the day. In addition they find it difficult to be heard in noisy situations. These symptoms lead often to an avoidance of social events with increased anxiety and frustration [4]. There are several studies that correlate dysphonia with a reduced voice related quality of life (VRQoL) [5,6]. Elderly patients in a better overall health condition demonstrate less perturbation (indicated by jitter and shimmer) and larger phonation ranges than subjects of comparable age, but reduced health status [7].

Vocal changes result from a number of alterations including changes of the larynx itself (calcification of the cartilaginous structures and joints, increased collagen content of laryngeal mucosa, muscular atrophy) but also from decreased pulmonary function, reduced mucosal secretions and diminished neuromotor control [8]. A large retrospective study identified vocal fold (VF) atrophy as the most prevalent finding in an elderly cohort [9]. The noticeable glottal gap and VF bowing are the most prominent video-laryngoscopic findings in these patients and are related to the atrophy of the laryngeal mucosa and the subjacent thyroarytenoid muscle (TAM) [10]. This glottal gap is the reason for air loss during phonation leading to a breathy voice quality. A large human cadaver study found that fiber diameters of the TAM decrease progressively over the course of aging from the 3^rd^ to the 9^th^ decade, independent of gender [10].

Changes in muscular VF tension and reduced viscoelastic properties are another cause for decreased vocal endurance. Studies have correlated changes in muscular function and voice parameters: Subglottal pressures were found to be similar in different age groups, but electromyographic signals were significantly lower in older individuals (esp. in the thyroarytenoid muscles [TAM]) [11]. Age related changes in laryngeal muscle electrical activity correlated with similar changes in other skeletal muscles. Diminished neural input is a possible reason for age-related muscle atrophy, as shown in the rat model. Significant changes were observed in the architecture of the neuro-muscular junction in the TAM between young, old and denervated muscle specimens [12]. These included a reduction in axonal terminal area and an increased number of postsynaptic receptor areas unoccupied by nerve terminals. The findings observed in the older muscles were similar to those found in the denervated muscles.

Current treatment of presbyphonia comprises voice therapy and surgical methods. While the first is based on the assumption of targeting the biological function of the presbylarynx, the latter restores passively the glottal competence and alters the natural laryngeal anatomy. Both aim at increasing vocal loudness, reducing vocal effort and increasing voice-related quality of life. Voice therapy requires several sessions with speech language pathologists which represent additional costs and is not available everywhere. Furthermore permanent practice by the patients themselves is needed, which most cannot sustain. Even though smaller studies have shown promising results [13], no randomized controlled trial has been performed. As the structures of interest (mainly TAM) are practically inaccessible in humans, outcome measures are limited to voice perception and stroboscopic ultrasound imaging. In other words, no conclusions about changes on the muscular level can be drawn. An ideal treatment would respect and preserve as far as possible the natural anatomy of the larynx and its afferent nerves and vessels.

Our study seeks to reverse the changes of laryngeal sarcopenia by specific chronic electrical nerve stimulation in an animal model. This approach is targeting the most prominent feature of the presbylarynx, the muscular glottal gap, on a cellular basis without damaging the larynx and its surrounding structures.

## Materials and Methods

### Animals

As described previously [14–16], we employed female sheep approximately 9 years of age as an aged animal model (breed: merino mountain sheep). Age was confirmed according to teeth status. Perioperative management and anesthesiologic procedures were carried out by the veterinarians at the Medical University of Graz. All procedures were approved by the Austrian Federal Ministry of Science, Research and Economy (approval number: BMWFW-66.010/0039-WF/V/3b/2015) and complied with the institution’s animal care guidelines. Sheep were kept in designated rooms in enclosures that are appropriate for the respective species. The animals had access throughout the day to an external weather proof stall and to the adjacent sheep meadow. Food and water were available *ad libitum* before surgery. All sheep were considered healthy based on clinical examination before the study.

All surgical interventions were made under general anaesthesia. All sheep were pre-medicated with midazolam (0.2 mg/kg), and ketamine (5 mg/kg). Anaesthesia was induced by using propofol (4 mg/kg). General anaesthesia was maintained with sevoflurane in oxygen, delivered from a standard circle rebreathing system and fentanyl in a continuous rate infusion at 20 μg/kg/hr. i.v. as well as propofol 2mg/kg/hr. An oesophageal cannula was placed in all animals. Intraoperative monitoring comprised pulse-oximetry, ECG, permanent measurement of body temperature and blood pressure. Intraoperative ventilation was performed with a PEEP of 5.6 cm H_2_O, with a total volume of 12.15 ml/kg.

Postoperative analgesia was performed four days after surgery with carprofen 4 mg/kg once daily and buprenorphine 0.01 mg/kg twice daily. Antibiotic treatment included gentamycin 4 mg/kg and penicillin 30.000 IU/kg for five days. Tetanus antitoxin (3000 I.U. IM) was given once postoperatively for prophylaxis. Body weight of animals was monitored once per week and did not change over the course of the experiment. For euthanasia an intravenous injection of thiopental (50 mg/kg) followed by T61 (Merck, 10 ml i.v.) was given in deep general anaesthesia.

### Determination of functional electrical stimulation parameters evoking fatigue of laryngeal muscles

To determine suitable functional electrical stimulation (FES) parameters for the laryngeal muscles, we performed acute experiments in four sheep. Hypertrophy of skeletal muscles is known to be achieved with FES settings that involve modest overload of the muscles [17]. In the case of the VF muscles we cannot readily measure or control the load against which they operate. Thus, a contraction design that results in muscular fatigue might provide an alternative.

Anaesthetized animals were tracheostomised and ventilated over a tracheostoma. The recurrent laryngeal nerve (RLN) was exposed unilaterally to enable stimulations by a cuff electrode (Ardiem Medical, PA, USA) (placed around the RLN ~15 cm below the thyroid cartilage). Contractions of laryngeal muscles were recorded by transnasal endoscopy. Paniello et al. described the laryngeal adductory pressure (LAP) as the pressure that is induced as the VF squeeze on a balloon while the RLN is stimulated [18]. Thus, we positioned a percutaneous transluminal angioplasty (PTA) catheter (balloon diameter 14mm, length 60mm; Clada Medical Devices, Galway, Ireland) between the VF to trace the pressure induced by distinct patterns of stimulation. The stimulation protocol consisted of fatigue producing sequences followed by a period of recovery and then non-fatiguing control bursts to verify that previous muscle activation did not influence the subsequent adductor contractions. The lowest current amplitude at which changes in the LAP could be observed was defined as the adductor muscle twitch threshold. We initially set the stimulation burst frequency to 20 Hz, where activity of the slow-twitch abductor posterior cricoarytenoid muscle (PCAM) predominates [19]. We could not detect a clear abduction movement here. Increasing the burst frequency resulted in incomplete fusion of the laryngeal adductor muscles activity, producing vocal fold adduction. As contractions elicited by stimulation frequencies above the fusion frequency appeared smooth [20], we subsequently increased the frequency in 10 Hz steps until no ripple could be observed in the pressure responses during contractions.

Fatiguing sequences varied in the stimulation duration, the pause-time between bursts, and the stimulation frequency (for examples, see S1 Fig). Quantitative analysis of the tested FES patterns was performed by calculation of the pressure-time integral (PTI), defined as the area under the pressure curve produced by a stimulation burst. The PTI responses for each pattern were normalized to the first contraction of the fatiguing sequence. A stable level of fatigue was reached if PTI did not decline with successive evoked contractions.

### Chronic RLN stimulation experiment—implantable pulse generator

The implantable pulse generator (IPG), or implant, was developed at the Centre for Medical Physics and Biomedical Engineering, Medical University of Vienna. The IPG (version label MiniVstim12B) was encapsulated by epoxy resin and had an outer diameter of 19 mm, a weight of 3.6 g and a volume of 2.5 cm³. A cuff electrode (same type as used for the acute experiments, Ardiem Medical, PA, USA) was directly connected to the IPG by an electrode lead with a length of 250 mm. The central, ring shaped electrode contact of the cuff was connected to the cathode and both lateral contacts to the anode of the IPG. The IPG was powered by a single lithium primary cell and generated monophasic constant current pulses followed by a charge balancing exponential reverse current. The device was freely programmable by a bidirectional radio frequency link between a programming device and the IPG. Stimulation patterns were designed, stored and modified on an android powered tablet computer, which was linked via Bluetooth to the programming device. Complex training (conditioning) patterns could be realized using a combination of predefined stimulation blocks and pause blocks. These predesigned training patterns were automatically delivered and repeated daily once the IPG was programmed.

**Fig 1:**
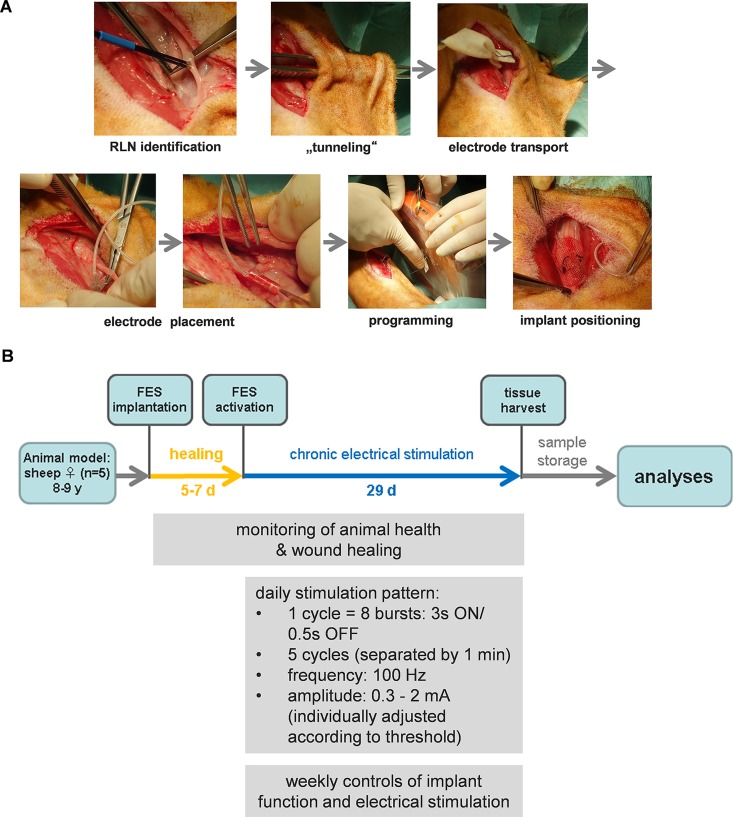
A. Surgical procedure for FES device implantation. B. Overview of chronic electrical stimulation experiment

For implantation surgery a lateral skin incision was made in the skin of the neck and the vascular nerve sheet containing vagal nerve, carotid artery and internal jugular vein were lateralized. The RLN was identified visually and by direct electrical stimulation with an insulated needle (Fig 1A). A cuff electrode was wrapped carefully around the RLN. Special care was taken not to touch the nerve itself with any instrument. Once in place, the electrode with the implant attached was tunnelled subcutaneously and sutured to the connective tissue adjacent to the ipsilateral sternocleidomastoid muscle. Wound closure was performed in layers.

An overview of the experimental procedures is presented in Fig 1B. After a healing period of 5–7 days the implants were activated. During the training phase the RLN was stimulated once every 24 hours always at the same time with 5 sets of contractions separated by one minute. Each set consisted of eight contractions (repetitions, 3s. ON/0.5s. OFF). Stimulation frequency was 100Hz, pulse width 256μs., initial amplitude 0.2–3mA. The total stimulation time was therefore only two minutes per day, or 0.14% of total time. The initial amplitude was adjusted for each sheep to three times the value that gave the first detected response obtained during the weekly test stimulations. Stimulation was maintained for 29 days.

### Harvest of laryngeal muscles

Larynges were excised immediately after euthanasia and the posterior cricoarytenoid muscles were exposed. For histological analyses, a sample from the central region of approximately 4 x 8mm in size was excised and mounted on a cork disc using Optimal Cutting Temperature (O.C.T.) compound (Scigen, Gardena, CA, USA). The sample was snap frozen in 2-methylbutane that was precooled by liquid nitrogen and then transferred into liquid nitrogen until the harvest of all larynges was completed. For RNA isolation and gene expression analysis an adjacent sample was excised and snap frozen in liquid nitrogen. Subsequently, larynges were opened ventrally by a cut through the midline. A sample from the central region of the TAM was excised and processed for histological analyses as described above. Finally, a small part of neighbouring TAM was excised and snap frozen in liquid nitrogen for RNA isolation and gene expression analysis. All samples were stored at -80°C.

### Triple immunofluorescence labelling of cryosections

Cryosections (10 μm) were prepared from frozen muscle samples and correct orientation of specimens was confirmed via standard haematoxylin and eosin staining. Subsequently, sections on Superfrost Plus microscope slides (Thermo Scientific, Waltham, MA, USA) were subjected to triple immunofluorescence labelling based on a previously described method [21]. Briefly, frozen cryosections were air dried for 30min. at room temperature before fixation with methanol/acetone (1:1) at -20°C for 15min. Slides were then rinsed three times for 5min. in phosphate-buffered saline (PBS) before incubation with rabbit anti-collagen 6 antibody (abcam, Cambridge, UK) at a dilution of 1:400 for 1h. After another rinsing step as described above, Alexafluor 488 goat anti rabbit diluted 1:1000 was added for 1h followed by a further rinsing step. Slides were then exposed to two different mouse monoclonal antibodies: (i) anti-type 1 myosin heavy chain MHY7 (Merk Millipore Corp., UK) directly labelled with Zenon 350 (Fisher scientific, UK) diluted 1:50, (ii) anti-type 2 myosin heavy chain MY32 (Abcam. Cambridge, UK) directly labelled with Zenon 594 diluted 1:1000. After incubation for 1h and washing as described above, sections were post fixed using 4% paraformaldehyde in PBS (15 min.) followed by a final PBS washing step and mounting in Vectashield mounting medium (Vector Laboratories, Peterborough, Cambridgeshire, UK). The sections were examined using a fluorescence microscope (Leica DM4000B microscope) with filters designed for each of the emitting wavelengths used. Slides were examined using a x10 objective and representative sections were selected by an experienced observer. These were chosen based on the presence of minimal artefacts, optimal sectioning of the muscle fibers, and relatively uniform tissue appearance. For each muscle, two representative images were captured using Axiovision software (Leica, Jena, Germany) and exported in.zvi format to Volocity software (PerkinElmer, Cambridge, UK) for background correction and conversion into grey level.tiff format ready for image analysis. Background correction used the blank images captured with the same objective for each filter block used. QWin Quips image analysis software V3 (Leica Microsystems, Heerbrugg, Switzerland) was then used to write a macro to count the fibers and measure the minimum feret diameter for each. The system was calibrated for the microscope and x10 objective used by measuring a slide graticule (1pixel = 1.01μm). The collagen grey level image was used to outline the border of each individual fiber and the minimum feret diameter measured. A total of 23,383 individual muscle fibers were measured and type classified in these five sheep, or on average 1169 per muscle analyzed. A linear mixed effects model was used to compare treatment and MFD and fibre type. A post-hoc comparison was performed with a Fisher-LSD and Bonferroni correction.

Percentage of collagen was calculated from the collagen 6 immunofluorescent images. Two random images from each muscle were captured using a Leica microscope x10 objective (Leica DM4000B) and AxioVision software. Background correction was performed using Volocity software. The corrected tiff images were used in Volocity to discriminate the pixels relating to collagen stain. The % collagen was calculated from the total pixel area of the 2 images for each muscle.

### RNA isolation, reverse transcription and quantitative PCR

For tissue homogenization, frozen samples (50–150 mg) were combined with MagNA Lyser Green Beads (Roche Diagnostics, Mannheim, Germany) and 700μL of QIAzol reagent (Qiagen, Hilden, Germany). Three homogenization steps (20sec at 6500rpm) were performed on the MagNA Lyser instrument with intermittent cooling of samples on ice for 1min. between Magnalyser runs. Subsequently, total RNA was isolated using the miRNeasy Mini kit (Qiagen, Hilden, Germany) according to the manufacturer’s instructions and stored at -80°C. RNA concentration was determined using a NanoDrop 2000c UV-Vis spectrophotometer (Thermo Scientific, Waltham, MA, USA). Quality of RNA was analysed using the 2100 Bioanalyzer system and the RNA 6000 Nano kit (Agilent Technologies, Frankfurt am Main, Germany). Specific RNA content was calculated by calculation of total RNA yield and normalization to the respective mass of muscle starting material. Reverse transcription (RT) of 1μg total RNA was performed using the QuantiTect Reverse Transcription kit (Qiagen, Hilden, Germany) according to the manufacturer’s protocol. Primer pairs for reverse transcription quantitative PCR (RT-qPCR) were designed using NCBI Primer BLAST; sequences are provided in supplementary S1 Table. RT-qPCR reactions were set up in 384-well plates (FrameStar® 480/384, 4titude, Berlin, Germany) using a Microlab STARlet device (Hamilton Robotics, Bonaduz, Switzerland). Reactions consisted of 5μL GoTaq® qPCR Master Mix (Promega, Mannheim, Germany), 1μL of the respective primer pair mix (each primer: 2μM; synthesized by microsynth, Balgach, Switzerland), and 4μL of the respective cDNA sample. Each combination of cDNA sample and primer pair (gene of interest) was pipetted in triplicate. RT-qPCR runs were performed on a LightCycler® 480 system (Roche, Vienna, Austria) using the following programme: 2min/95°C (denaturation), 45 cycles of 10sec/95°C and 1min/60°C (amplification), ramping at 2.5°C/min from 55°C to 95°C (melting curve analysis). Prior to analysis of the study samples, equal amounts of RNA from PCAM and TAM of three sheep were mixed to generate an “RT-“control that was used in subsequent runs, as well as a pooled cDNA sample which was used in a 5-step dilution series (1:5) to calculate the PCR efficiencies of individual primer pairs. The cDNA pool was further used at 2.5ng RNA/μL on every subsequent run to enable inter-plate calibration. Likewise, the working concentration of study cDNA samples for RT-qPCR was 2.5ng RNA/μL. After each run, the generation of a single type of amplicon per well was confirmed by melting curve analysis. Quantitation cycle (Cp) values were obtained using the AbsQuant/2^nd^ Derivative Max method of the LightCycler® 480 software and were further processed using the R packages “ReadqPCR” and “NormqPCR”. Cp values from technical triplicates were averaged. Several candidate internal reference RNAs were analysed in preliminary experiments using the geNorm algorithm [22], resulting in the identification of UXT and B2M as transcripts with the lowest variation in expression levels across samples (gene stability values M: 0.33 and 0.42, respectively). Transformation of Cp values to relative quantities (RQ) was performed by subtracting individual Cp values from a reference Cp value (originating from a single cDNA sample (sheep S11, left PCAM)–primer pair (B2M) combination) and incorporated the previously determined gene-specific efficiencies [23]. The geometric mean of relative quantities of UXT and B2M was subsequently used as normalization factor to obtain normalized relative quantities (NRQ) for all mRNAs of interest. Overall, this strategy enabled the relative comparison of mRNA levels between distinct samples, as well as an assessment of the relative abundances of distinct mRNAs within the same sample.

## Results

### Acute experiments

#### Assessment of laryngeal muscle fatigue—FES parameters

**Fig 2:**
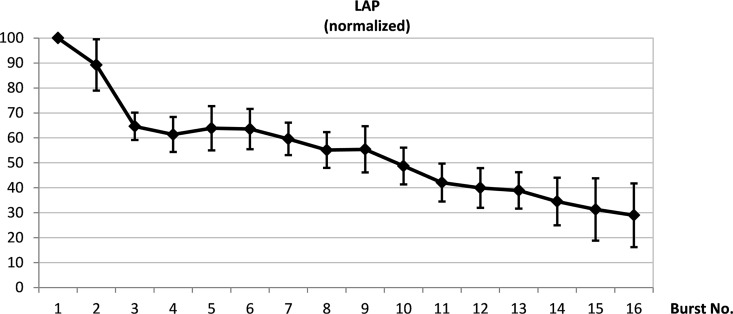
Determination of optimal electrical stimulation parameters. Representative traces of stimulation-induced contraction of laryngeal muscles, reflected by averaged pressure changes of a liquid-filled balloon that was positioned in the glottic rim. Decline in laryngeal muscle force relative to the first stimulation-induced contraction (n = 4).

For various stimulation patterns, the decline in the burst pressure area relative to the first burst was measured. The neuromuscular electrical stimulation fatigue protocol consisting of 16 bursts (3s. stimulation, 0.5s. pause-time) reduced the LAP by ~71% of the initial values. After the 10^th^ burst, the LAP was already reduced by more than 50% and the laryngeal adductor muscles were considered to have reached a stable level of fatigue. Fig 2 shows the drop in average normalized LAP as a function of the delivered stimulation burst.

### Chronic RLN stimulation

#### Healing and chronic electrical stimulation phases

Implantation surgery was well tolerated. No animal developed wound infection during the healing and chronic electrical stimulation phases. Sheep were monitored when the FES pattern was delivered for the first time; no visible signs of stress could be observed. All IPGs remained intact during the experiment, i.e. the training pattern was successfully delivered over the period of 29 days (Fig 1B), proper stimulation was confirmed endoscopically twice during the stimulation period under light sedation, as well as directly before euthanasia. Electrode impedances varied between animals (320Ω – 2200Ω after implantation), but remained approximately constant throughout the following weeks (S2A Fig). Stimulation thresholds, which were between 0.1mA and 0.6mA at the beginning, gradually increased over time, with a maximum difference of approximately 3-fold when first and last measurements were compared (S2B Fig).

#### Immunohistology

**Fig 3:**
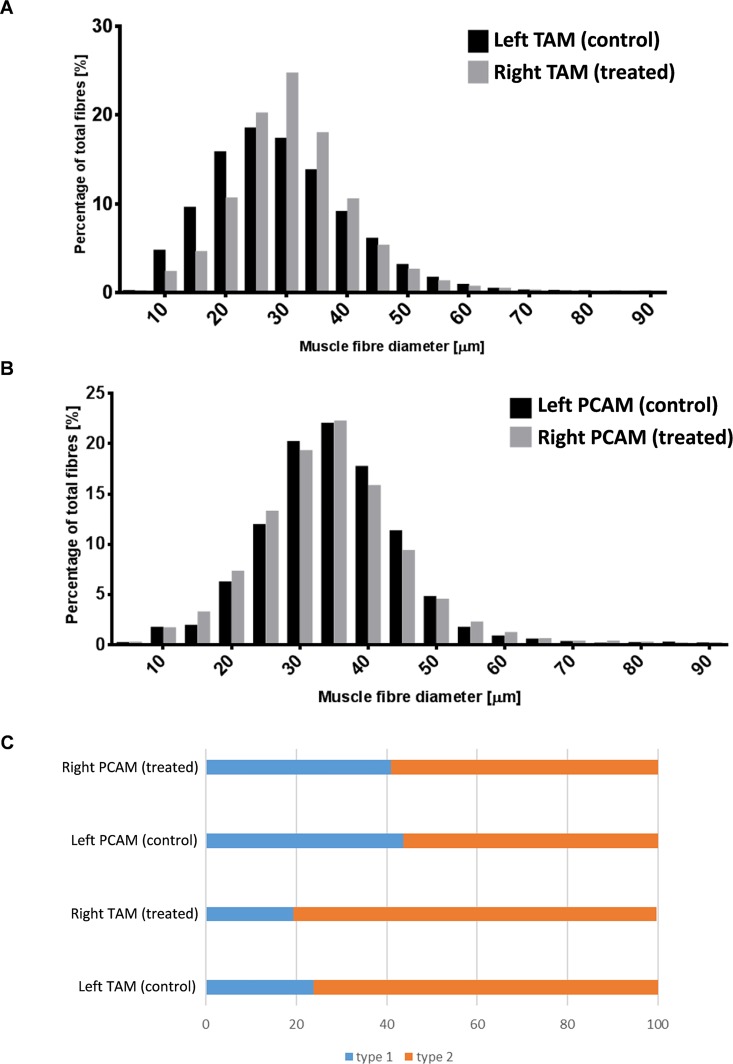
**A-B. Analysis of stimulation-induced changes in muscle fiber diameter (MFD) of TAM (A) and PCAM (B).** MFD values were grouped in 10 μm bins and are presented as percentage of total fibers **3C. Analysis of stimulation-induced changes in fiber type distribution**. Relative percentages of type 1 and type 2 fibres for both muscles.

Cryosections from right (stimulated) and left (unstimulated control) TAM and PCAM were stained immunohistologically from all animals. When type I and II fibers of the TAM were analysed as one group, we found a significant shift in mean fiber diameter (MFD) towards larger diameters in the stimulated side: The mean muscle fiber diameter of the stimulated (right) TAM was significantly larger than the non-stimulated (left) TAM (30μm±1.1μm vs. 28μm±1.1 μm, p<0.001; Fig 3A; p < 0.001; linear mixed effects model with Fisher-LSD and Bonferroni correction). There was a significant difference in mean fiber diameter of type 1 fibers between stimulated (27 μm±0.7μm) and non-stimulated TAM (25μm±0.7μm), as well as between type 2 stimulated (33μm±1.3μm) and non-stimulated (31μm±1.3μm) TAM muscle fibers (p<0.001).

No such differences could be detected in the PCAM (Fig 3B). Furthermore there was no shift in fiber type proportions between the stimulated and unstimulated side of either muscle, even if there was a significantly higher proportion of type I fibers in PCAM compared to TAM (p<0.001; Fig 3C). This was also true when comparing the relative fiber type distribution in TAM and PCAM of control animals. Content of collagen -as an indirect marker of muscle damage- did not differ between stimulated and unstimulated TAM and PCAM (S3 Fig).

#### Analysis of specific RNA content

**Fig 4:**
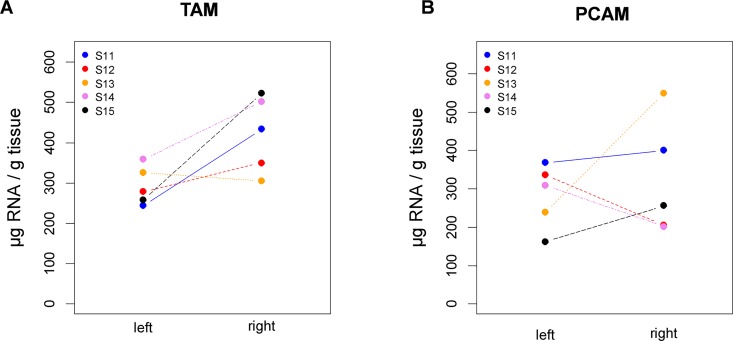
Changes in RNA concentration of laryngeal muscles induced by electrical stimulation. (A) Specific RNA yield for TAM. (B) Specific RNA yield for PCAM.

Skeletal muscle hypertrophy has been previously shown to be paralleled by increases in muscular RNA content [24]. Specific RNA content of TAM was increased in four of the five animals when comparing right (stimulated) versus left (un-stimulated) side (Fig 4A). This trend was not evident for PCAM (Fig 4B). So as with fiber diameter, TAM appeared to have a larger response than PCAM.

#### Quantitative PCR

**Fig 5:**
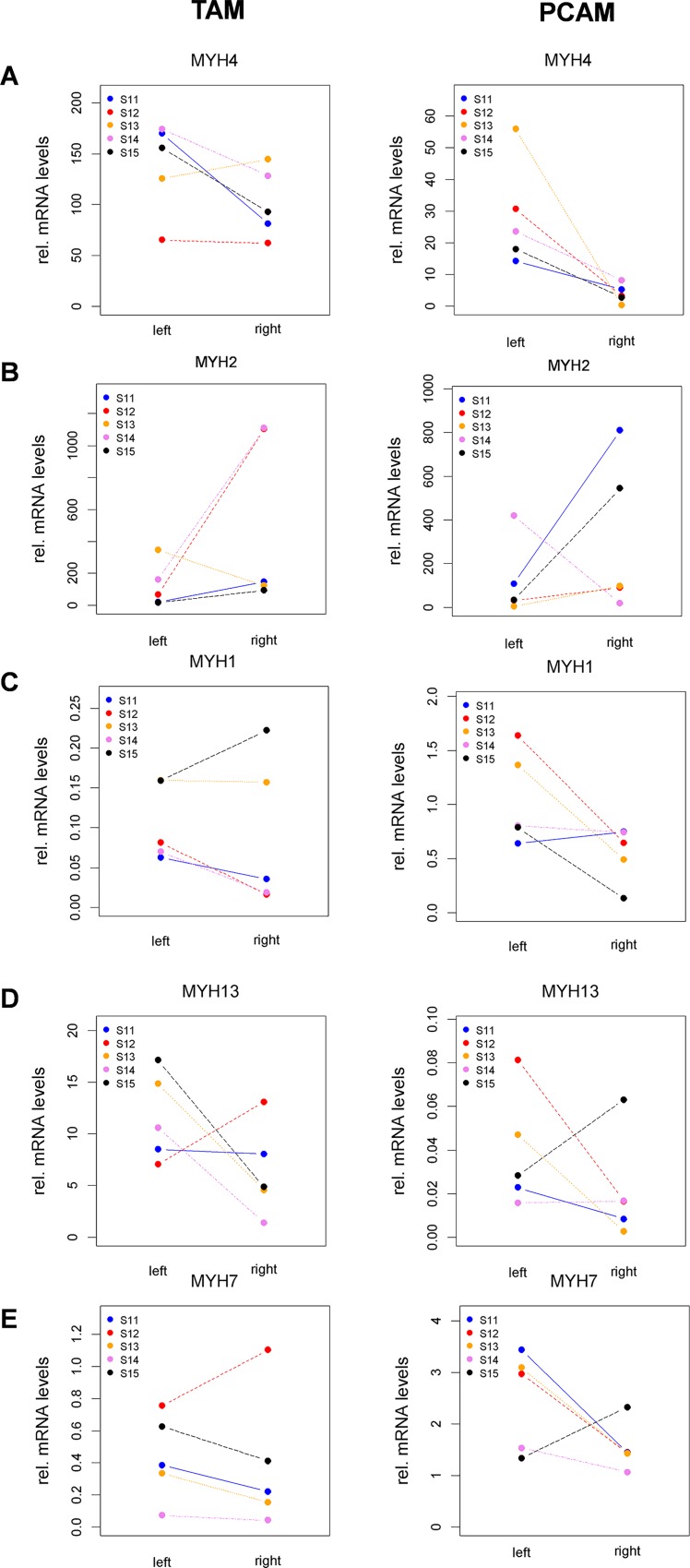
RT-qPCR analysis to quantify the expression of myosin heavy chain isoforms in left (unstimulated) and right (stimulated) TAM (left panels) and PCAM (right panels). (A) MYH4 (MyHC-IIb), (B) MYH1 (MyHC-IId/x), (C) MYH2 (MyHC-Iia), (D) MYH13 (MyHC-IIL), (E) MYH7 (MyHC-I).

We performed a comprehensive quantification of mRNAs coding for the five skeletal muscle myosin heavy chain (MHC) isoforms, i.e. type IIb (MYH4), type IId (MYH1), type IIa (MYH2), type II-eo (MYH13), and type I (MYH7). Analysis of the abundance of each isoform relative to other isoforms in the same muscle corroborated previous studies of laryngeal muscles, e.g. substantial expression of the fastest isoform type II-eo was detected only in TAM, and expression of the slow/oxidative isoform type I was higher in PCAM than TAM [25,26]. As FES might affect expression of MHC isoforms, we compared right (stimulated) versus left (un-stimulated) side. For PCAM, mRNA levels of the type IIb isoform were strongly decreased on the right side in all five animals (Fig 5A, right panel). In TAM, this decrease was obvious only in three animals (Fig 5A, left panel). For both PCAM and TAM, expression of the type IIa isoform was increased on the right side in four of the five animals (Fig 5B). No trends for a FES-induced effect could be observed for the other two fast isoforms type IId (Fig 5C) and type II-eo (Fig 5D). For both PCAM and TAM, expression of the slow type I isoform was modestly decreased on the right side in four of the five animals (Fig 5E).

We also analyzed mRNA levels of the key myogenic transcription factors myogenin (MYOG) and myogenic differentiation 1 (MYOD1), the satellite cell marker paired box 7 (PAX7), as well as two important regulators of mitochondriogenesis, peroxisome proliferator-activated receptor gamma coactivator 1-α (PPARGC1A, PGC-1α) and mitochondrial transcription factor A (TFAM). However, no clear FES-induced effects on mRNA abundance could be observed for any of these genes (S4 Fig).

## Discussion

As current treatment modalities for presbyphonia are often cost-intensive and unsatisfactory, there is a strong demand for novel approaches which can ameliorate age-related voice impairments. Reversing the atrophy of important laryngeal muscles (such as the TAM) by functional electrical stimulation (FES) is one such highly promising new strategy. In the present study we could show that FES in a single daily session led to a significant increase in mean muscle fiber diameter of the TAM in a large animal model in a short period of time with a very conservative conditioning pattern. We identified diameter increases of about 5% compared to the untreated contralateral side. The VF atrophy and the consequent glottal gap as the main laryngoscopic finding of the presbylarynx is caused mainly by an age related bilateral reduction of the TAM fiber diameters [10]. Unlike conventional treatment (voice therapy and phonosurgery), FES can provide a new treatment method that can target for the first time the affected muscle via its natural nerve fibers. Furthermore, the inevitable co-activation of the PCAM appeared to have no adverse effect on this opening muscle.

FES has been applied successfully to neuromuscular defects of the larynx in a number of animal trials, although the underlying laryngeal pathology was different (VF paralysis) [27–29]. In addition, the targeted muscle was different (PCAM instead of TAM) and frequently an intervention with acute RLN injury/denervation was made. Most of these studies showed promising results in terms of restoring abductory movement of the VF, improving ventilation parameters, and demonstrating the safety of the intervention [30]. However, choosing the right stimulation parameters, and the daily pattern of stimulation sessions requires considerable care and further research. McMullen et al. achieved an increase of neuromuscular junction density and mitochondrial content via electrical stimulation in a small rodent model, but essential parameters, such as mean muscle fiber diameter decreased [31]. In this case the stimulation represented 1.67% of total time whereas we used stimulation for 0.14% of total time. In contrast to our study animals were set into general anaesthesia for each stimulation, which can be very demanding especially for older animals. Furthermore, effects of stimulation on the larynx were not checked during the study period.

To induce muscle hypertrophy to counteract the age-related laryngeal muscle atrophy, muscle overload is required. Prescriptions for human exercise programs are often expressed in terms of the *1 repetition maximum* (RM). 1RM means the load that can be lifted only once without resting before another attempt, and is thus an indicator of maximum strength. The current recommendations for resistance exercise for human participants given by the American College of Sports Medicine [32] are expressed as rather wide ranges of repetitions and sets, but a typical recommendation is to use a load approximately 70% of 1RM, so that typically about eight contractions can be achieved before failure that demands a recovery period before another set can be attempted.

In the case of the VF muscles, the load against which they operate cannot be readily measured or controlled, neither can 1RM be estimated. Designing a type of contraction that results in some fatigue can be an appropriate approach here. It is a reasonable assumption that the amount of fatigue, assumed in sets to failure of approximately eight repetitions, is about 20–30%. In contractions that activate every motor neuron, sets that produce approximately this level of loss of muscular response within eight repetitions can be arranged. Laryngeal muscle contractile function was assessed by measuring the pressure inside a PTA balloon placed within the VF. While this is clearly an indirect measurement, we found that sets of fused contractions elicited by 100Hz stimulation for 3s with a 0.5s pause produced fatigue sufficient to reduce the functional output (the area under the pressure curve) by about 30% after eight contractions. We therefore chose a stimulation pattern that follows quite closely the recommendations for resistance training in humans, except that stimulation was delivered once every day instead of once every two or three days.

In line with previous studies in humans and other animal models, our immunohistological and PCR-based analyses revealed a marked difference in MHC fiber type distribution between TAM and PCAM. Compared to PCAM, TAM had a higher relative amount of fast (and superfast) fibers and thus a lower amount of slow fibers, reflecting the muscle’s distinct functional requirements. We could not detect by immunocytochemistry significant changes in fiber type distribution between the stimulated and control side. This finding was corroborated as FES did not induce significant changes in the mRNA levels of TFAM (promoting mitochondrial DNA replication [33]), and PGC-1α, a master regulator of fiber type switching [34]. Although exercise can result in a “fast-to-slow” fiber type transition [35,36], it should be noted that our experimental design involved the relatively short training period of 29 days. Further, we employed a rather conservative FES pattern, i.e. the daily training session lasted only 7.5 min with a total of 2 min of tetanic contraction. Thus, while we observed some differences in MHC mRNA expression levels between stimulated and control sides, these differences might mark the very early phase of muscular adaptation to a training pattern which, if prolonged, might also evoke shifts in fiber type distribution. Lastly, the fact that size distribution of TAM fibers was significantly shifted towards larger diameters is most important with respect to both the pathology (sarcopenia) and the envisioned treatment.

Aging does not affect muscle fiber types equally but has a greater impact on MHC II fibers than type I fibers [37]. Although data for intrinsic laryngeal muscles is scarce, it is known that there is a trend to decreased type II and increased type I fibers in TAM with increasing age [38]. Interestingly there were considerably different manifestations of FES in adductory (TAM) versus abductory (PCAM) muscles. Significant changes in mean fiber diameter were not detected in the PCAM, but only in the TAM. We know from studies in aged rodents that muscles with adductory functions (phonation and airway protection) are more impaired than muscles with abductory functions (respiration) [39].

In summary, we have employed sheep as a preclinical model to test the power of FES as a means to reverse age-related laryngeal muscle atrophy. Acute fatigue measurements of laryngeal adductor muscles led to the identification of a favourable FES pattern which was subsequently used as training protocol in a chronic setting. This is the first study that shows that FES is safe way to reverse age-related shrinking of laryngeal muscle fiber diameter. Unlike other approaches, radio frequency controlled implants allow controlled and progressive application of stimulation and, if necessary, adaptation of training programs without repetitive anaesthesia. Following studies will explore implantation techniques more suitable for transfer into the human setting as well as additional and more effective stimulation patterns.

## Supporting Information

S1 Fig Determination of optimal electrical stimulation parameters. (A,B) Exemplary traces of stimulation-induced contraction of laryngeal muscles, reflected by pressure changes of an air-filled balloon that was positioned in the glottic rim. Depending on the duration of stimulation, the pause-time between stimulation, and the frequency, (A) no fatigue or (B) fatiguing of laryngeal adductor muscles was observed.
(TIF)

S2 Fig (A) Traces of electrode impedances at the time of FES device implantation and subsequent weekly follow-up controls. (B) Traces of lowest current amplitudes (thresholds) for stimulation-induced contraction of laryngeal muscles at the time of FES device implantation and subsequent weekly follow-up controls.
(TIF)

S3 Fig Analysis of relative abundance of collagen in TAM and PCAM. (A) Immunofluorescent staining and subsequent analysis of microscopy images were employed to obtain the percentage of collagen V-immunoreactive area for left (control) and right (treated) sides. (B) Representative images from fluorescent microscopy for PCAM and TAM.
(TIF)

S4 Fig RT-qPCR analysis to quantify the expression of (A,B) the myogenic transcription factors Myogenin (MYOG) and myogenic differentiation 1 (MYOD1), (C) the satellite cell marker Paired box 7 (PAX7), and (D,E) the mitochondiral markers peroxisome proliferator-activated receptor gamma, coactivator 1 alpha (PPARGC1A) and mitochondrial transcription factor A (TFAM) in left (unstimulated) and right (stimulated) TAM (left panels) and PCAM (right panels).
(TIF)

S1 Table RT-qPCR primer sequences. (PDF)
