# Correction: Reversing Age Related Changes of the Laryngeal Muscles by Chronic Electrostimulation of the Recurrent Laryngeal Nerve

**DOI:** 10.1371/journal.pone.0172660

**Published:** 2017-02-16

**Authors:** Michael Karbiener, Jonathan C. Jarvis, Justin D. Perkins, Hermann Lanmüller, Martin Schmoll, Hanna S. Rode, Claus Gerstenberger, Markus Gugatschka

The affiliation for the sixth author is incomplete. Hanna S. Rode is affiliated with Department of Veterinary Clinical Sciences, Royal Veterinary College, London, United Kingdom and Institute of Veterinary Anatomy, Histology and Embryology, Faculty of Veterinary Medicine, University of Leipzig, Leipzig, Germany.
