# Supplementary figures and images for: Reversing Age Related Changes of the Laryngeal Muscles by Chronic Electrostimulation of the Recurrent Laryngeal Nerve
Source: PLoS One. 2016 Nov 28;11(11):e0167367. doi: 10.1371/journal.pone.0167367 (PMC5125708; doi:10.1371/journal.pone.0167367)

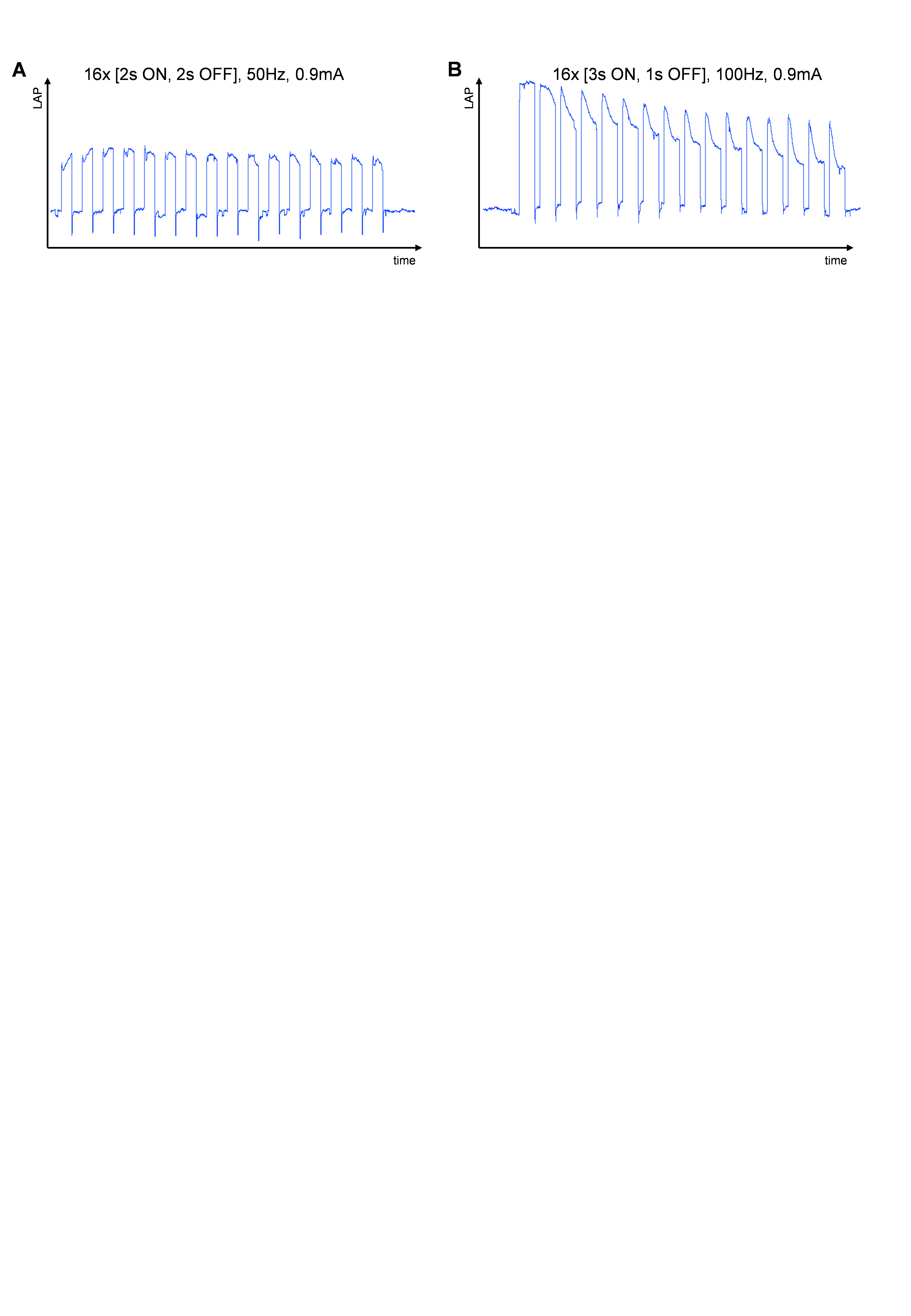

Supplement: S1 Fig — (A,B) Exemplary traces of stimulation-induced contraction of laryngeal muscles, reflected by pressure changes of an air-filled balloon that was positioned in the glottic rim. Depending on the duration of stimulation, the pause-time between stimulation, and the frequency, (A) no fatigue or (B) fatiguing of laryngeal adductor muscles was observed. (TIF) [file pone.0167367.s001.tif]

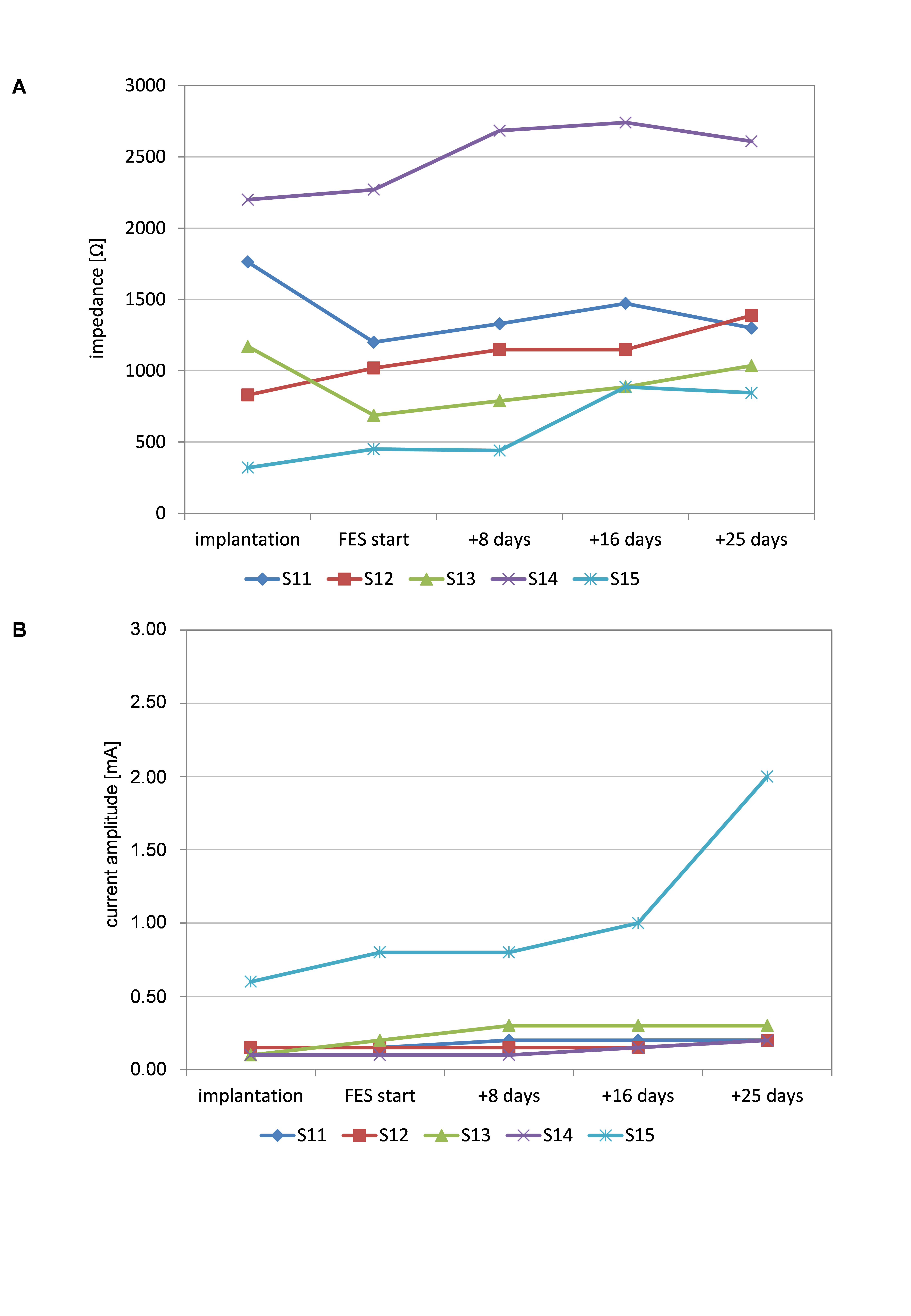

Supplement: S2 Fig — (A) Traces of electrode impedances at the time of FES device implantation and subsequent weekly follow-up controls. (B) Traces of lowest current amplitudes (thresholds) for stimulation-induced contraction of laryngeal muscles at the time of FES device implantation and subsequent weekly follow-up controls. (TIF) [file pone.0167367.s002.tif]

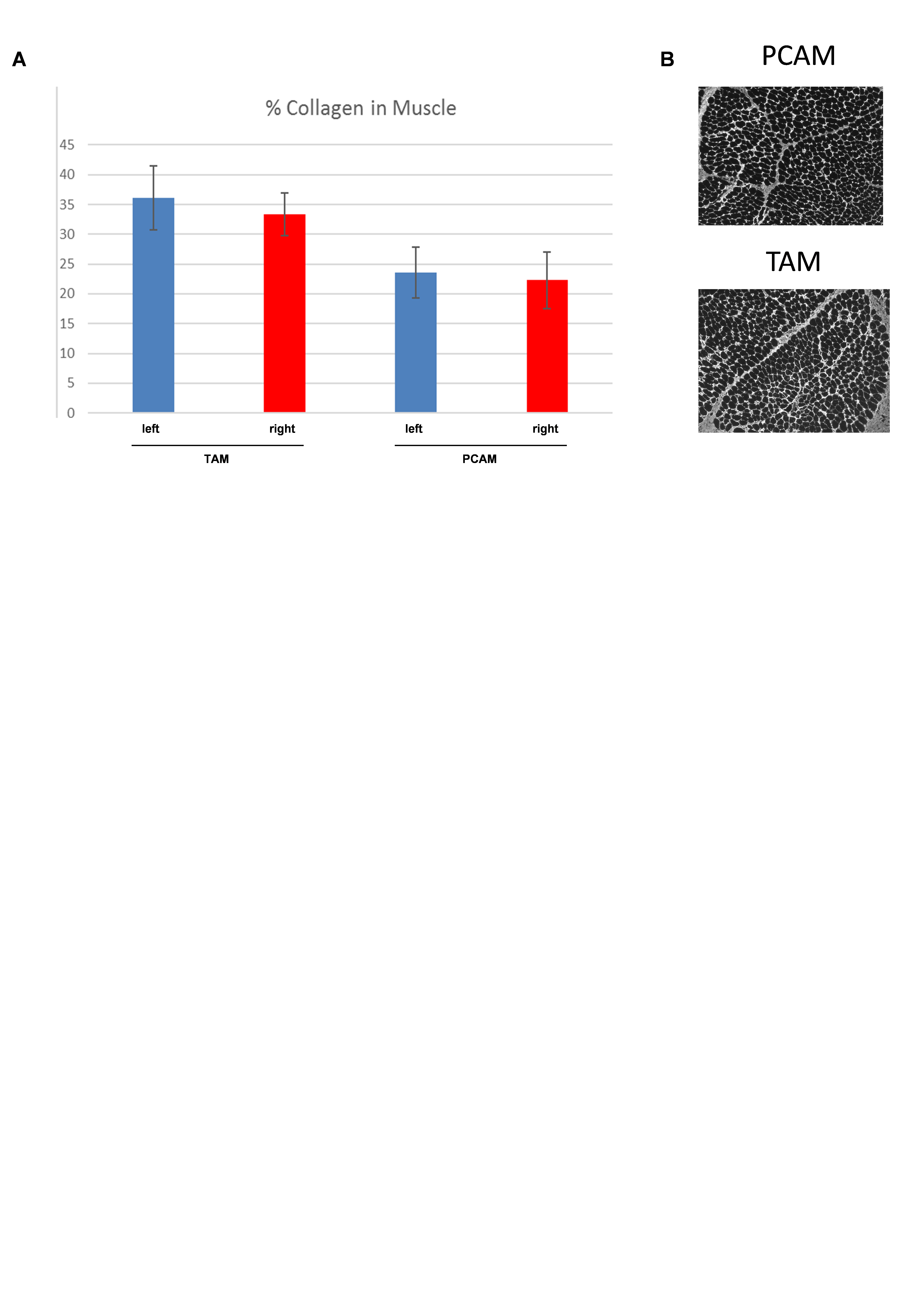

Supplement: S3 Fig — (A) Immunofluorescent staining and subsequent analysis of microscopy images were employed to obtain the percentage of collagen V-immunoreactive area for left (control) and right (treated) sides. (B) Representative images from fluorescent microscopy for PCAM and TAM. (TIF) [file pone.0167367.s003.tif]

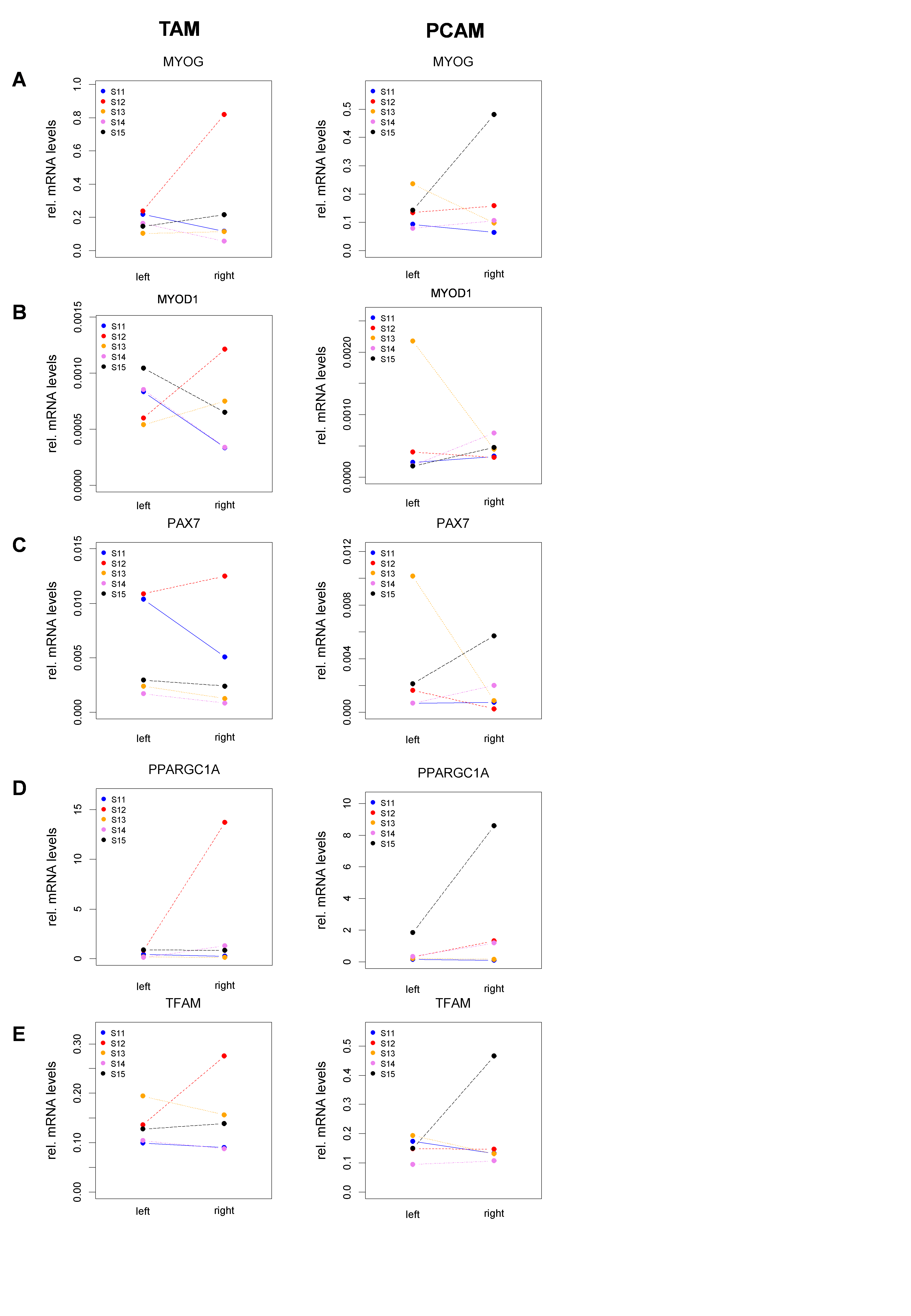

Supplement: S4 Fig — RT-qPCR analysis to quantify the expression of (A,B) the myogenic transcription factors Myogenin (MYOG) and myogenic differentiation 1 (MYOD1), (C) the satellite cell marker Paired box 7 (PAX7), and (D,E) the mitochondiral markers peroxisome proliferator-activated receptor gamma, coactivator 1 alpha (PPARGC1A) and mitochondrial transcription factor A (TFAM) in left (unstimulated) and right (stimulated) TAM (left panels) and PCAM (right panels). (TIF) [file pone.0167367.s004.tif]
