# Supplementary material for: Reversing Age Related Changes of the Laryngeal Muscles by Chronic Electrostimulation of the Recurrent Laryngeal Nerve
Source: PLoS One. 2016 Nov 28;11(11):e0167367. doi: 10.1371/journal.pone.0167367 (PMC5125708; doi:10.1371/journal.pone.0167367)
Supplement: S1 Table — (PDF) [file pone.0167367.s005.pdf]

| gene symbol | also known as | NCBI Reference Sequence | forward primer (5'-3') | reverse primer (5'-3') |
|---|---|---|---|---|
| UXT | | XM_012183356.1 | GACTCCAGGAAGCTAATCACTC | TGAGAGCTTCTGCCAGTGT |
| B2M | | NM_001009284.2 | GCCATCCAGCGTATTCCAGA | CCCCGTTCTTCAGCAAATCG |
| LOC101111980 | MYH4 | XM_004012702.2 | CTGCAAGACTTGGTGGACAA | TGGAGTTTGCGGAATTTGGA |
| LOC442994 | OMYHC2X, MYH1 | XM_004012706.3 | GTTCTCTGGCGCAGCATCT | GAGTGCTCCTCAGGTTGGTC |
| LOC101103165 | MYH13 | XM_012185735.2 | TGCAGAAGCAGGCGACTC | TTTCCCTGAACACAGCGGAC |
| LOC443471 | OMYHC2A, MYH2 | XM_015098654.1 | AATGGCAGTCTTTGGGGAGG | AAAGATTCCTTGGGCTCGGC |
| MYH7 | OMYHCS | XM_004010325.2 | TGCTGACAGACAGAGAAAACCAG | TTTTGCTGCGGTCGCCAAT |
| MYOD1 | | NM_001009390.1 | CTCCGCGACGTAGACTTGAC | CAGATCCGGGGAGTCGAAAC |
| MYOG | | NM_001174109.1 | TCAGCTCCCTCAACCAGGAG | GACTGCAGGAGGCACTATGG |
| PAX7 | | XM_012157510.1 | CCGTGCCCTCAGGTTTAGTG | CCCTTTGTCGCCCAGGATG |
| PPARGC1A | PGC1; PPARGC1 | XM_012179733.2 | AAGGCAATTGAAGAGCGCCG | AGCTGTCTCCATCATCCCGC |
| TFAM | | XM_015104510.1 | AGCTCAAAACCCAGATGCAAAA | TATACCTGCCAGTCTGCCCT |
